# Higher percentage of CD133^+ ^cells is associated with poor prognosis in colon carcinoma patients with stage IIIB

**DOI:** 10.1186/1479-5876-7-56

**Published:** 2009-07-07

**Authors:** Chun-Yan Li, Bao-Xiu Li, Yi Liang, Rui-Qing Peng, Ya Ding, Da-Zhi Xu, Xin Zhang, Zhi-Zhong Pan, De-Sen Wan, Yi-Xin Zeng, Xiao-Feng Zhu, Xiao-Shi Zhang

**Affiliations:** 1 State Key Laboratory of Oncology in South China, Cancer Center, Sun Yat-sen University, Guangzhou, PR China; 2 Biotherapy Center, Cancer Center, Sun Yat-sen University, Guangzhou, PR China; 3 Department of Abdominal Oncology, Cancer Center, Sun Yat-sen University, Guangzhou, PR China; 4 Department of Experimental Research, Cancer Center, Sun Yat-sen University, Guangzhou, PR China; 5 Biotherapy Center, The First Affiliated Hospital, Chongqing Medical University, Chongqing, China; 6 GuangZhou First Municipal People's Hospital, Guangzhou, China

## Abstract

**Background:**

Cancer stem cell model suggested that tumor progression is driven by the overpopulation of cancer stem cells and eradicating or inhibiting the symmetric division of cancer stem cells would become the most important therapeutic strategy. However, clinical evidence for this hypothesis is still scarce. To evaluate the overpopulation hypothesis of cancer stem cells the association of percentage of CD133^+ ^tumor cells with clinicopathological parameters in colon cancer was investigated since CD133 is a putative cancer stem cell marker shared by multiple solid tumors.

**Patients and methods:**

Tumor tissues matched with adjacent normal tissues were collected from 104 stage IIIB colon cancer patients who were subject to radical resection between January, 1999 to July, 2003 in this center. The CD133 expression was examined with immunohistochemical staining. The correlation of the percentage of CD133^+ ^cell with clinicopathological  parameters and patients' 5-year survival was analyzed.

**Results:**

The CD133^+ ^cells were infrequent and heterogeneous distribution in the cancer tissue. Staining of CD133 was localized not only on the glandular-luminal surface of cancer cells but also on the invasive budding and the poorly differentiated tumors with ductal structures. Both univariate and multivariate survival analysis revealed that the percentage of CD133^+ ^cancer cells and the invasive depth of tumor were independently prognostic. The patients with a lower percentage of CD133^+ ^cancer cells (less than 5%) were strongly associated with a higher 5-year survival rate than those with a higher percentage of CD133^+ ^cancer cells (greater than or equal to 55%). Additionally, no correlation was obtained between the percentage of CD133^+ ^cancer cells and the other clinicopathological parameters including gender, age, site of primary mass, pathologic types, grades, and invasive depth.

**Conclusion:**

The fact that a higher percentage CD133^+ ^cells were strongly associated with a poorer prognosis in patients with locally advanced colon cancer implicated that CD133^+ ^cancer cells contribute to the tumor progression, and the overpopulation hypothesis of cancer stem cell seems reasonable.

## Background

Colorectal cancer is one of the most common causes of cancer death worldwide. Although the median overall survival of patients with metastatic colorectal cancer has increased from 12 months to approximately 24 months over the past decade as a result of an improvement in systemic therapies including new chemotherapeutic agents such as CPT-11 and oxaliplatin and monoclonal antibodies against EGFR (cetuximab and panitumumab) and VEGF (bevacizumab), the 5-year survival is still pessimistic [1-4]. Therefore, one of the main challenges in colorectal carcinoma remains to develop new strategies beyond chemotherapy to inhibit the disease progression.

A growing body of evidence supports the notion that only a small subset of cells within a solid tumor have 'stem-like' characteristics. These tumor-initiating cells, or cancer stem cells, distinct from non-malignant stem cells, show low proliferative rates, high self-renewal capacity, propensity to differentiate into active proliferating tumor cells, and resistance to chemotherapy or radiation [5,6]. Until now, cancer stem cells have been identified in a great deal of solid tumors [5-8].

Multiple cancer stem cell-associated markers have been identified, among which CD133 has received considerable attention. CD133 or prominin-1 gene is located on chromosome 4p15.32 and encodes a cell surface glycoprotein compromising five transmembrane domain and two large glycosylated extracellular loops [9,10]. The transcription of CD133 can be initiated at five tissue specific promoters, yielding eight alternatively spliced transcripts [11-13]. Epigenetic mechanism is involved in the regulation of CD133 expression [14-16]. Although the function of CD133 is unknown, preliminary evidence proposed that expression of CD133 is associated with the activation of stemness-related signal pathway, resistance to apoptosis and bioenergetic stress [17-22]. Initially identified in hematopoietic stem cells, CD133 is now shared as cancer stem cell marker across multiple kinds of solid tumors, such as those in the brain, breast, lung, liver, colon, prostate, pancreatic carcinomas, medulloblastoma, and melanoma [5-7,23-29].

As for colorectal cancer, initially, Ricci-vitiani and O'Brien observed that colon cancer stem cells are located in the CD133^+ ^subpopulation, which accounts for approximately 2.5% of the tumor cells [30,31]. Subsequently, Dalerba and Haraguchi reported that markers for colon cancer stem cells are EpCAM ^hi^/CD44^+^/CD166^+ ^[32,33]. In addition, Barker proposed that Lgr5 is another marker [34]. CD133^+ ^colon cancer cells include EpCAM ^hi^/CD44^+ ^cells, whereas the relationship between CD133^+ ^subset and Lgr5^+ ^subset is unclear. Therefore, which protein would be an ideal marker for colorectal cancer stem cells remains an open question.

Based on the mathematic model, the hypothesis that development of colorectal carcinoma is driven by overpopulation of stem cells has been suggested. It is believed that the abundance of cancer stem cells is derived from their symmetric division, whereas their normal partners are subject to asymmetric division, therefore, eradicating or inhibiting the symmetric division of cancer stem cells would become the most important strategy for cancer treatment [35-39]. If the percentage of cancer stem cells is associated with the prognosis of cancer patients, the overpopulation hypothesis would be substantially supported. By now, the relationship between the percentage of CD133 and prognosis of colorectal carcinomas was controversial. Horst reported that CD133 expression is an independently prognostic marker whereas this kind of correlation was not confirmed by Kojima[40,41]. Accordingly, more evidence was need to elucidate the relationship between the percentage of CD133^+ ^tumor cells and the prognosis of colorectal cancer patients. This study showed that the percentage of CD133^+ ^tumor cells was associated with the prognosis among patients with locally advanced colon cancers, implicating that CD133^+ ^cells are involved in the progression of colon cancer.

## Patients and methods

### Patients and Follow-up

104 cases of pathologically confirmed specimens were obtained from colon carcinoma patients with TNM stage IIIB (the depth of primary invasive spread defined as T3 and T4 with one to three regional node involvement but no distant metastasis) who were subject to radical resection between January, 1999 and July, 2003 in the Cancer Center of Sun Yat-Sen University, Guangzhou, China. None of the patients had undergone either chemotherapy or radiotherapy before the collection of the samples. All of them were subject to 5-Fu based postoperatively adjuvant chemotherapy for six months. Patients were observed on an every-three-month basis during the first year, once every 6 months in the second year, and by telephone or mail communication once every year thereafter for a total of 5 years. If recurrence or metastasis occurred, 5-Fu based chemotherapy was given according to the NCCN guideline. Overall survival was defined as the time from operation to death or was censored at the last known alive data. Histopaothologic characteristics were confirmed by blinded review of the original pathology slides. The TNM classification was used for pathologic staging, and the World Health Organization classification was used for pathologic grading.

### Immunohistochemical assay

The expression of CD133 in primary tumors matched with adjacent noncancerous tissue was examined with immunohistochemical assay. Briefly, formalin-fixed, paraffin-embedded archived tissues were subject to 4-μm section. Then, sections were subject to dewax, rehydration, blocking with hydrogen peroxide, and antigen retrieval with microwave in a 10 mM citrate buffer (pH 6.0) for 10 min and cooled to room temperature. After being blocked with 1% goat serum albumin sections were incubated with the mouse monoclonal antibodies against human CD133 at a dilution of 1:150 (Abcam, Cambridge, UK) overnight at 4°C, followed with horseradish peroxidase-labeled secondary antibodies for 30 minutes at room temperature. The sections were developed with diaminobenzidine tetrahydrochloride (DAB) and counterstained with hematoxylin. Immunohistochemical assay was performed within 7 days of section preparation. To prevent antigen degradation sections were stored at 4°C before immunohistochemical analysis. Tissue derived from glioma was used as positive control and negative controls were made with primary antibody replaced by PBS.

Referring to Maeda's method, slides were examined under low power (×40 ~ ×200) microscope to identify the regions containing the highest percentage of CD133^+ ^cells (hot spot) in the cancer nest [42]. Ten fields of hot spot inside the tumor tissue were selected, and expression of CD133 was evaluated in 1000 tumor cells (100 cells per field) with high power (×400) microscopy. Specimens were defined as positive for CD133 expression if there were tumor cells distinctly stained by the anti-CD133 antibodies. The percentage of CD133^+ ^cells was classified into two levels: < 5% CD133-positive cells and ≥ 5% CD133-positive cells[42].

### Statistical analysis

The following factors were assessed with both univariate and multivariate analyses for their influence on overall survival: gender, age (<60 years old vs ≥ 60 years old), sites of primary mass (left hemicolon vs right hemicolon), the T stages (the depth of primary invasive spread, T3 vs T4), pathological classifications (papillary carcinoma and tubular adenocarcinoma vs mucoid adenocarcinoma and signet ring cell carcinoma), tumor grades (the degree of cellular differentiation, well differentiated, G1 vs moderate differentiated, G2 vs poorly differentiated, G3), and the percentage of the CD133^+ ^cells (<5% positive vs ≥5% positive). The nonparametric correlation Kendall's tau-b test was used to assess associations between categorical variables. Kaplan-Meier curves were used to estimate the distributions of clinicopathological  characteristics to survival and compared with the log-rank test. The Cox regression model was used to correlate assigned variables with overall survival. All statistical analyses were carried out using SPSS 15.0 software (SPSS Inc, Chicago, IL, USA). Statistical significance was assumed for a two-tailed *P *< 0.05.

## Results

### Expression of CD133 in tumor tissue

**Figure 1:**
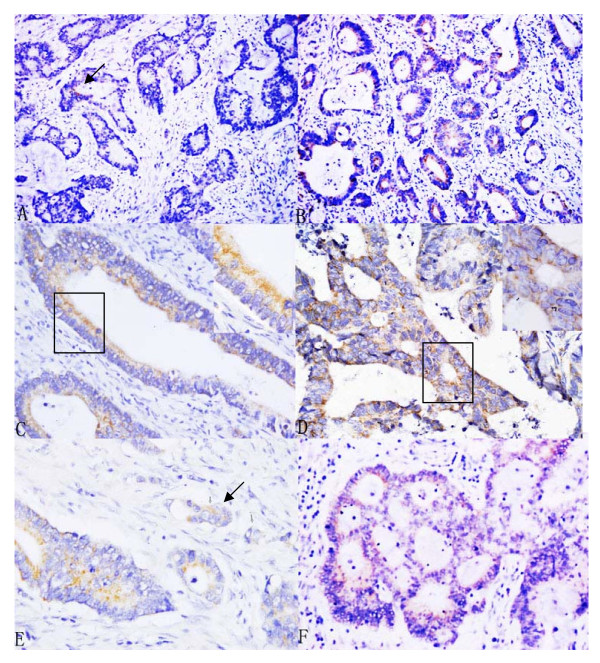
**The expression of CD133 in colon cancer patients with stage IIIB (10 × 20~10 × 100)**. The expression of CD133 was examined with immunohistochemical assay. (A): <5% CD133^+ ^cells in the cancer nest; (B): ≥5% CD133^+ ^cells in the cancer nest; (C) and (D): the staining of CD133 on the luminal surface and the basal surface of cancer cells; (E): the staining of CD133 on budding cancer nest; (F): the staining of CD133 on poor-differentiated cancer nests with ductal structures.

CD133 brownish signals were observed on the cell membrane, especially on its luminal and basal surface. In general, the cases with intensive staining of CD133 had higher percentage of CD133^+^ tumor cells. Several nests with intensive CD133 staining, so-called "hotspot" could always be seen within the field of cancer nests microscopically (Fig 1A to 1D). The cancer cells within an adenocarcinoma nest could actively proliferate and form a group of cells, which invaded into the surrounding tissue, so-called "budding", and showed negative or weak staining against CD133 (Fig 1E). Besides staining on the well differentiated tumors CD133 staining was documented on the poorly differentiated tumors with ductal structures rather than those without ductal structures (Fig 1F). The paratumorous normal intestinal epithelium could be found in 72 out of 104 specimens used for this study. The CD133 expression of normal intestinal epithelium was only found in 7 out of the 72 specimens.

Referring to Maeda's method the percentage of CD133^+ ^cells was classified into two levels: < 5% CD133^+ ^cells and ≥ 5% CD133^+ ^cells [39]. In this group of patients, 62 cases of 104 (59.6%) specimens contained less than 5% CD133-positive tumor cells and 42 cases (40.4%) contained more than 5% CD133-positive tumor cells, among which the percentage of CD133^+ ^cells varying from 5% to 25% existed in 23 cases, from 26% to 50% in 12 cases, and more than 50% in 7 cases.

### Relationship between the percentage of CD133^+ ^cells and clinicopathological characteristics

No correlation was observed between the expression of CD133 and clinicopathological parameters such as age, gender, sites of primary mass, pathological classifications, invasive depth, and tumor grades. Otherwise, the analysis revealed that mucoid adenocarcinomas and signet ring cell carcinomas had the potential with poorer differentiation (r = 0.459, *P *< 0.001) and higher frequency occurred in the right hemicolon (r = 0.215, *P *= 0.022) (Tab 1).

**Table 1:** Correlations of CD133 expression with clinicopathological parameters in the Stage IIIB colon carcinomas

Variables		gender	age	Invasive depth	Sites of primary mass	Grades	Pathological classifications	The percentage of CD133^+ ^cells
gender	P	.	.242	.541	.792	.129	.129	.785
age	P	.242	.	.312	.075	.455	.869	.249
Invasive depth	P	.541	.312	.	.895	.272	.426	.499
Sites of primary mass	P	.792	.075	.895	.	.936	.022*	.786
Grades	P	.129	.455	.272	.936	.	.000**	.536
Pathological classifications	P	.129	.869	.426	.022*	.000**	.	.333
The percentage of CD133^+ ^cells	P	.785	.249	.499	.786	.536	.333	.

*: *P *< 0.05; **: *P *< 0.001

### Relationship between survival and clinicopathological characteristcs assessed with univariate survival analysis

By the end of the 5-year follow-up, 67 cases were still alive. So, the 5-year survival rate was 64.4%. Kaplan-Meier analysis revealed that the percentage of CD133^+ ^cells in cancer nests and the invasive depth of primary mass were prognostic. The 5-year survival rate among patients with a higher percentage of CD133^+ ^cells (≥5%) in the cancer nests was 45.2%, whereas those with a lower percentage of CD133^+ ^cells (<5%) was 77.4% (*P *= 0.001). In addition, the 5-year survival rate among patients with T3 tumors (tumors which invade through the muscular propria into the subserosa, or into nonperitonealizd pericolic tissue) was 69.6%, whereas the 5-year survival rate among patients with T4 tumors (tumors which perforate the visceral peritoneum or directly invade other organs or structure) was 25.0% (*P *= 0.001)(Tab 2).

**Table 2:** Assessment of overall survival for stage IIIB colon carcinoma patients by clinicopathological  parameters with univariate and multivariate analysis

Clinicopaothological characteristics	N (n = 104)	5-year survival	Kaplan-Meier analysis *P *value	Cox regression model analysis *P *value
		64.4%		
Gender			0.461	0.479
male	65	61.5%		
female	39	69.2%		
		64.4%		
Age			0.148	0.211
≥ 60 year old	57	57.9%		
<60 year old	47	72.3%		
		64.4%		
Sites of primary mass			0.291	0.381
Left hemicolon	60	68.3%		
Right hemicolon	44	59.1%		
		64.4%		
Pathological classifications			0.423	0.139
papillary + tubular	82	65.9%		
mucoid + signet ring	22	59.1%		
		64.4%		
Grades			0.154	0.114
G1	5	60%		
G2	80	68.8%		
G3	19	47.4%		
		64. 4%		
Invasive depth			0.001	0.002
T3	92	69.6%		
T4	12	25.0%		
		64.4%		
The percentage of CD133^+ ^cells			0.001	0.004
≥5% CD133 positive	42	45.2%		
<5%CD133 positive	62	77.4%		

### Relationship between survival and clinicopathological characteristics assessed with multivariate survival analysis

**Figure 2:**
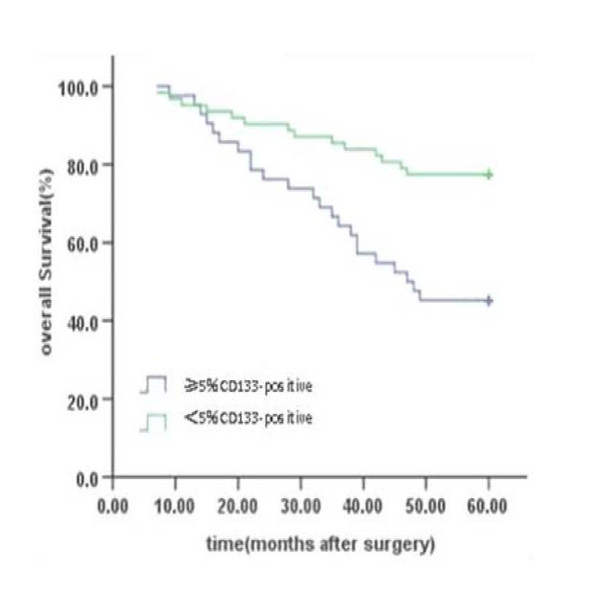
**The association of overall survival with the percentage of CD133+ cells in colon carcinoma patients with Stage IIIB**. The patients with a lower percentage of CD133^+ ^cells (<5%) in the cancer nests were strongly associated with longer 5-year survival than those with a higher percentage of CD133^+ ^cells (≥5%).

The Cox regression model revealed that the patients with a lower percentage CD133^+ ^cells (<5%) in the cancer nests were significantly associated with a higher 5-year survival rate with -0.987 in partial regression coefficient and 0.373 (95% CI 0.190 ~ 0.732) in relative risk (*P *= 0.004). Additionally, a higher T stage (invasive depth) was significantly associated with a lower survival rate with 1.209 in partial regression coefficient and 3.351 (95% CI 1.558 ~7.208) in relative risk (*P *= 0.002). Therefore, the percentage of CD133^+ ^cells in cancer nests and T stage were independently prognostic factors. No relationship was observed between the survival and the other clinicopathological parameters such as age, gender, site of primary mass, pathological classifications, and grades (Tab 2, Fig 2).

## Discussion

This study showed that a higher percentage of CD133^+ ^cells in cancer nests was strongly associated with the lower 5-year survival rate in colon cancer patients with stage IIIB, a locally advanced disease among which most of patients would die from metastasis in spite of adjuvant chemotherapy, implying that the overpopulation hypothesis of cancer stem cell seems reasonable as CD133 is a putative marker of colon cancer stem cells.

The evidence concerning the correlation of the percentage of CD133^+ ^tumor cells with the prognosis of patients was scarce as a few of observations were reported [43-46]. Recently the relationship between CD133 expression and prognosis in colorectal carcinomas was examined. Horst reported that CD133 expression is an independently prognostic marker whereas this kind of correlation was not observed by Kojima. [40,41] The discrepancy might derived from inadequate patient quantity and the mixed tumor stage. For example, in Kojima's study, a total of 189 patients consisted of 106 cases of colon cancers and 83 cases of rectal cancers with TNM stages varying from I to VI, that is, one group of patients with a definite stage contained only 20 or 30 cases of colon or rectal cancer patients, respectively[41]. Similar situation existed in Horst's study [40]. To narrow the heterogeneity of patients and make the results more reproducible this study included 104 cases of colon carcinoma patients with stage IIIB. The results showed that CD133^+ ^cancer cells contributed to the progression of colon cancer, arguing the Hosrt's observation.

The discrepancy concerning the pattern and the frequency of CD133 expression in colon cancer also existed between the studies mentioned above and this study. Horst and Kojima reported that CD133 antigen, stained with antibodies from Miltenyi Biotech, Sata Cruz Biotechnology, or Cell signaling, was localized exclusively on the glandular-luminal surface of colorectal cancer. Staining of the CD133 was observed neither on the budding cancer nest nor on poorly differentiated cancer cells [40,41]. However, in this study, being stained with antibodies from Abcam CD133 expression existed not only on the apical membrane but also on basal surface of tumor cells, both on the budding cancer nest (the invasive front) and on the poorly differentiated cancer cells, although the intensity of staining was weaker. This pattern of CD133 expression might be more likely consistent with the hypothesis that CD133^+ ^cancer cells would reveal a more aggressive phenotype. Since the intensity of CD133 is cell cycle-dependent, among which the least CD133 immunoreactive cells are in the G0/G1 portion, and the increased CD133^+ ^cells is correlated with increased DNA content, and cancer cells is relatively arrested in the invasive front, so, attenuated expression of CD133 occurred in the invasive front (budding)[47,48]. As for the frequency of CD133^+ ^cells in colorectal cancers the discrepancy also existed. In Kojima's study CD133 expression was detected in only 29 of the 189 tumors (15.3%). Of these, 21 tumors (11.1%) showed CD133 over-expression among which CD133 positive area occupied more than 10% of the entire tumor tissue[41]. Otherwise, in Horst's study tumors with more than 50% of CD133^+ ^tumor cells exist in 20 out of 79 colorectal cancers (25.3%) [40]. In this study, the percentage of CD133+ cells varying from 5% to 25% existed in 23 cases (22.1%), from 26% to 50% in 12 cases (11.5%), and more than 50% in 7 cases (6.7%). Therefore, it is reasonable to infer that the heterogeneous patterns and frequencies of CD133 expression in colon cancer derived from the specificity of antibody clones used. In the future, more attention should be paid to the specificity of CD133-targeting antibodies, the standardization of the CD133 positive cells classification system, and homogeneity of tissues.

Recently the representative of CD133 as marker of colon cancer stem cells was questioned. On the one hand, CD133^+ ^colon cancer cells revealed 'stem-like' characteristics, and stem cells marked by CD133 was susceptible to transformation into tumors[49]. On the other hand, CD133 expression was detected not only on cancer cells, but also on the luminal layer of epithelium of digestion duct, on the mature epithelium of the pancreatic duct, on the proximal tubules of the kidney, and on the lactiferous ducts of the mammary gland [50-52]. Furthermore, both CD133^+ ^and CD133^- ^metastatic colon cancer cells initiated tumors[50]. Additionally, CD44^+ ^cancer cells rather than CD133^+ ^cells have an increased tumorigenicity[53]. Those data pointed that CD133 should not be a unique marker for colon cancer stem cells. It is less likely that a known marker for colon cancer stem cells, such as CD44, CD166, EpCAM, and Lgr5, has the potential just like Pten-related pathway in leukemia, which could distinguish hematopoietic stem cells from leukemia-initiating cells [54-57]. Collectively, a combination of cell surface markers is need for the definition of colon cancer stem cells [58-60]. This study implied that, given that CD133 may not represent all the entire cancer stem cells, it is still a useful biomarker as CD133^+ ^cells is more aggressive than CD133^- ^partners in colon cancer.

## Conclusion

The fact that a higher percentage of CD133^+ ^cells is strongly associated with a poorer prognosis implicates that CD133^+ ^cells contribute to the progression of colon cancer, and the overpopulation hypothesis of cancer stem cell seems reasonable.

## Competing interests

The authors declare that they have no competing interests.

## Authors' contributions

XDZ, PRQ, DY, ZX, PZZ, and WDS carried out the cases collection, LCY and LY carried out the immunohistochemical staining work, LBX and ZXF analyzed results. ZXS and ZYX conceived of the study, participated in its design and coordination and helped to draft the manuscript. All authors read and approved the final manuscript
